# Preserved Ejection Fraction Despite Progressive Functional Decline in a Patient With Single-Ventricle Physiology Without Documented Successful Surgical Palliation

**DOI:** 10.7759/cureus.115087

**Published:** 2026-08-24

**Authors:** Mohamad Karaali, Elias Hanna, Georges Ghanem, Walid Tarcha

**Affiliations:** 1 Internal Medicine, Lebanese American University Medical Center, Beirut, LBN; 2 Cardiology, Lebanese American University Medical Center, Beirut, LBN

**Keywords:** adult congenital heart disease (achd), atrial fibrillation (af), chronic hypoxemia, fontan palliation, preserved ejection fraction, pulmonary hypertension, single ventricle physiology

## Abstract

Seventh-decade survival with single-ventricle congenital heart disease (CHD) without documented successful surgical palliation is exceedingly rare. The prognostic value of ejection fraction (EF) in this population remains poorly characterized, and validated staging tools are absent from current guidelines.

We report a 62-year-old man with complex single-ventricle CHD who survived without documented successful surgical palliation and had a common atrium, systemic right ventricle, and severe common atrioventricular valve regurgitation. Despite a preserved EF of 55-60%, he was NYHA Class IV with chronic hypoxemia. Four hospitalizations over 26 months were driven by atrial fibrillation with rapid ventricular response and community-acquired pneumonia; medication nonadherence was a documented precipitant in at least two admissions. He died during the fourth admission of refractory hypoxemic respiratory failure.

In this patient, preserved EF did not reflect functional reserve in the context of single-ventricle physiology. AV valve regurgitation, obligate right-to-left shunting, and preload dependence may collectively decouple systolic function from effective cardiac output. Arrhythmia and infection were the dominant modifiable tipping points in this case. This case illustrates the potential inadequacy of EF-centered staging and the critical role of medication adherence, arrhythmia management, and infection prevention in deferring irreversible functional decline.

## Introduction

Single-ventricle congenital heart disease (CHD) encompasses a heterogeneous group of cardiac malformations characterized by a functionally single ventricular chamber [1]. The global birth prevalence of CHD is approximately 9 per 1,000 live births, with complex lesions, including single-ventricle physiology, accounting for a significant proportion of CHD-related morbidity and mortality [1]. Without surgical palliation via the Fontan procedure, survival beyond the fourth decade is rare, and seventh-decade survival is exceedingly uncommon [2,3]. The Fontan circulation, established through staged palliation, directs systemic venous return directly to the pulmonary circulation, bypassing the single ventricle. In the small subset of patients who survive without palliation, physiological adaptation, including balanced pulmonary-to-systemic flow, compensatory polycythemia, and chronic hypoxemia, may sustain life for decades, though at significant functional cost [2,3].

Ejection fraction (EF) remains a commonly used metric for assessing ventricular function in clinical practice; however, its utility as a prognostic surrogate in single-ventricle physiology is poorly established [2,3]. We report a 62-year-old man with single-ventricle CHD without documented successful surgical palliation who maintained preserved EF (55-60%) yet deteriorated to New York Heart Association (NYHA) Class IV across four hospitalizations over 26 months, dying of refractory respiratory failure. This case illustrates that preserved systolic function may not preclude end-stage decline in unpalliated single-ventricle physiology. It highlights the diagnostic and therapeutic challenges encountered when managing complex CHD without access to a dedicated adult CHD (ACHD) specialist, a reality for the majority of such patients worldwide [2,3].

## Case presentation

A 62-year-old man presented with a two- to three-week history of productive cough and progressive dyspnea. His home SpO₂ had dropped to 62% on 10 L of oxygen via face mask the preceding day. He had self-discontinued bisoprolol and digoxin one week prior to admission.

**Figure 1:**
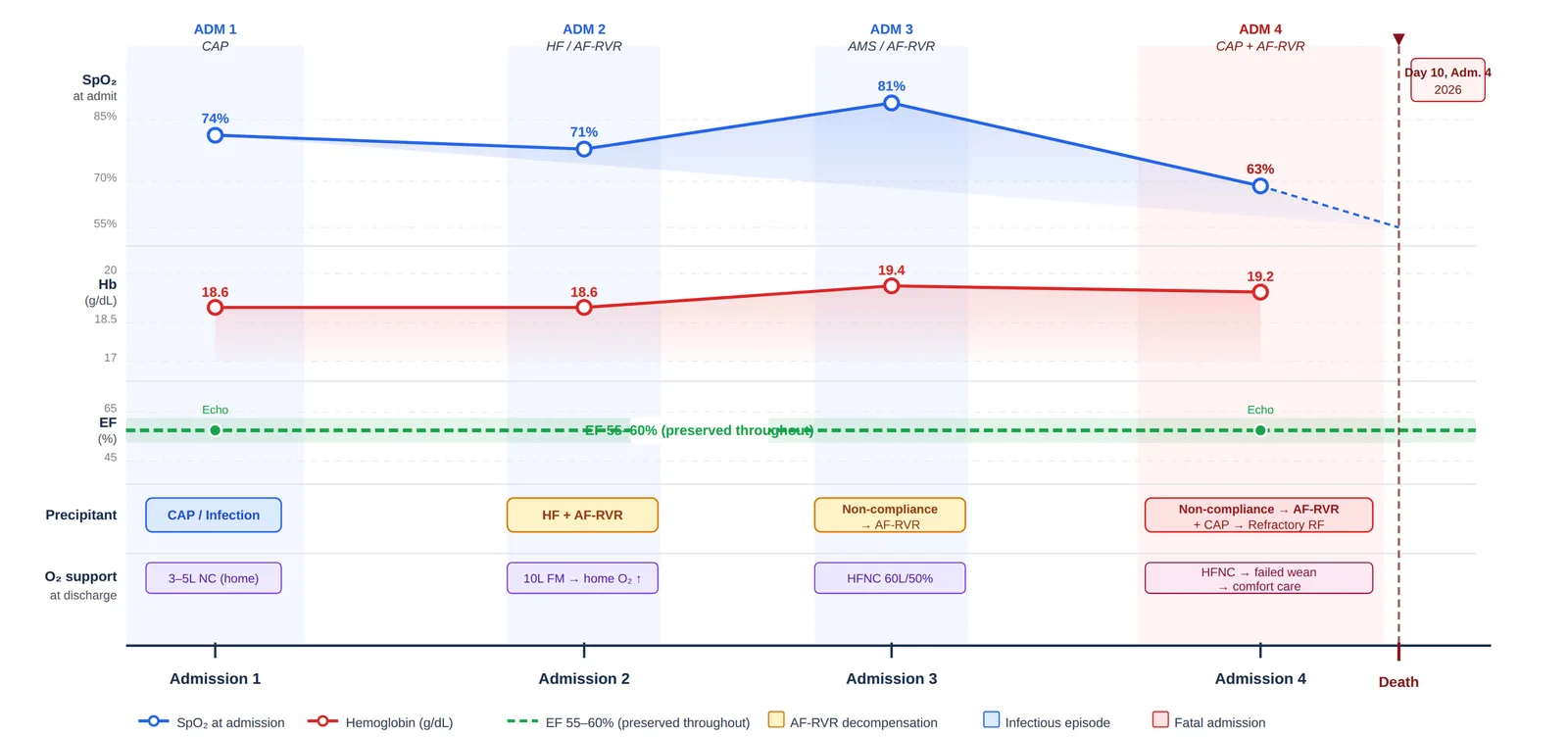
Longitudinal clinical trajectory across four hospital admissions illustrating the dissociation between preserved EF and progressive functional decline in single-ventricle physiology without documented successful surgical palliation ADM: admission, AF-RVR: atrial fibrillation with rapid ventricular response, CAP: community-acquired pneumonia, EF: ejection fraction, HFNC: high-flow nasal cannula, HF: heart failure, Hb: hemoglobin, NC: nasal cannula, O₂: supplemental oxygen, RF: respiratory failure, SpO₂: peripheral oxygen saturation

On arrival, SpO₂ was 63% on a 15 L face mask, heart rate was 130 bpm (irregular), blood pressure was 120/80 mmHg, and the patient was afebrile. Examination revealed bilateral crackles, a holosystolic murmur along the sternal borders, digital clubbing, and facial plethora from polycythemia. There was no lower extremity edema. This was his fourth hospitalization in 26 months (Table 1; Figure 1).

**Table 1:** Summary of four hospitalizations AKI: acute kidney injury, AMS: altered mental status, AF: atrial fibrillation, CAP: community-acquired pneumonia, CTA: computed tomography angiography, CXR: chest X-ray, EF: ejection fraction, FM: face mask, HFNC: high-flow nasal cannula, INR: international normalized ratio, NC: nasal cannula, PE: pulmonary embolism, RVR: rapid ventricular response, SpO₂: peripheral oxygen saturation, TSH: thyroid-stimulating hormone

Admission	Presentation	Key findings	Management
Admission 1	1-month productive cough; SpO₂ 74%	CAP; elevated INR 3.2; CXR right-sided pleural thickening; TSH 70 mIU/L, free T4 not available in records	IV ceftriaxone + azithromycin; bisoprolol initiated; amiodarone discontinued
Admission 2	Progressive dyspnea; orthopnea; SpO₂ 71% on 3 L NC	PaO₂ 41 mmHg; AF with RVR; left pleural effusion; no PE on CTA	IV furosemide 40 mg BID; digoxin loading
Admission 3	AMS; somnolence; home HR 180 bpm	AF with RVR 130–160 bpm; AKI Stage I; possible lacunar infarct on CT brain	IV digoxin + bisoprolol; electrolyte correction; antibiotics deferred
Admission 4	SpO₂ 62% at home on 10 L FM; medications self-discontinued for 1 week	AF with RVR (HR 130s bpm); LLL collapse + bilateral pleural effusions; Hb 19.2 g/dL; CRP 21 mg/L	IV ceftriaxone + azithromycin; bisoprolol + digoxin reinstated; HFNC; comfort care

Past medical history

The patient carried a diagnosis of complex single-ventricle CHD without documented successful surgical palliation: a common atrium with severely deficient interatrial septation, systemic right ventricle, mesocardia with situs solitus, and severe common atrioventricular (AV) valve regurgitation. Precise segmental classification could not be established from available historical records. A cardiac surgical attempt in the 1990s was unsuccessful; a concurrent right lower lobectomy resulted in chronic pleural thickening and restrictive lung disease. He remained unpalliated thereafter. Complete anatomical classification, including great artery relationships and coronary anatomy, could not be established. Dedicated cardiac CT or MRI was never performed. The dominant echocardiographic impression was a single ventricular chamber with a common AV valve, most consistent with an unbalanced AV canal with dominant right ventricular morphology; a double-outlet right ventricle could not be excluded. Cardiac position was mesocardia with situs solitus on all echocardiographic reports. A prior echocardiogram noted a circular atrial image that possibly represented residual surgical hardware from the 1990s attempt. EF was assessed qualitatively in technically limited studies; formal biplane quantification was not feasible due to anatomical complexity and suboptimal acoustic windows. Three echocardiograms are available: the 2010 and 2011 studies described preserved systolic function without a specific EF value; the 2024 study formally reported an EF of 55-60%. The reason for palliation failure was unknown.

No dedicated ACHD specialist was involved; management was provided by internal medicine and general cardiology, with pulmonology consultation. Comorbidities included chronic hypoxemia, secondary polycythemia (hemoglobin, 18-19.4 g/dL), permanent atrial fibrillation (AF) treated with acenocoumarol, a vitamin K antagonist used in preference to direct oral anticoagulants because of the lack of established evidence supporting their use, heart failure with preserved systolic function (NYHA Class IV), amiodarone-induced hypothyroidism, and chronic kidney disease. Rate control was achieved with bisoprolol and digoxin. The patient had three prior hospitalizations for pneumonia, heart failure, and AF with rapid ventricular response (RVR).

Differential diagnosis

The differential diagnosis included (1) community-acquired pneumonia with hypoxemic respiratory failure (supported by productive cough, elevated CRP, and CT findings of left lower lobe collapse); (2) heart failure decompensation from AF with RVR (supported by medication noncompliance and prior similar admissions); (3) pulmonary embolism (considered low probability given prior negative CT angiography and clinical presentation; formal exclusion on current admission was not pursued). The clinical picture was consistent with pneumonia-triggered respiratory failure superimposed on AF with RVR.

Investigations

Laboratory studies showed a hemoglobin level of 19.2 g/dL, WBC count of 9.62 × 10⁹/L, neutrophils of 75.4%, CRP level of 21.1 mg/L, creatinine level of 1.23 mg/dL, international normalized ratio (INR) of 2.18, lactic acid level of 0.5 mg/dL, and sodium level of 134 mEq/L. Viral testing was negative. Arterial blood gas analysis on admission while receiving oxygen via a 15-L face mask showed a pH of 7.38, PaCO₂ of 42 mmHg, PaO₂ of 44 mmHg, HCO₃⁻ of 25 mEq/L, and oxygen saturation of 76%, consistent with hypoxemic respiratory failure.

ECG confirmed AF with RVR. A chest CT demonstrated left lower lobe subsegmental collapse and bilateral pleural effusions; findings were interpreted as consistent with an infective process in the clinical context, though these findings are not specific for infection. Cardiomegaly was noted. Echocardiography (admission 1) confirmed EF 55-60% with severe common AV valve regurgitation and elevated filling pressures (right atrial pressure ~20 mmHg; dilated inferior vena cava without inspiratory collapse), technically limited by structural complexity. Outflow tract anatomy and right ventricular outflow tract morphology could not be characterized on any available echocardiographic study due to severely limited acoustic windows.

**Figure 2:**
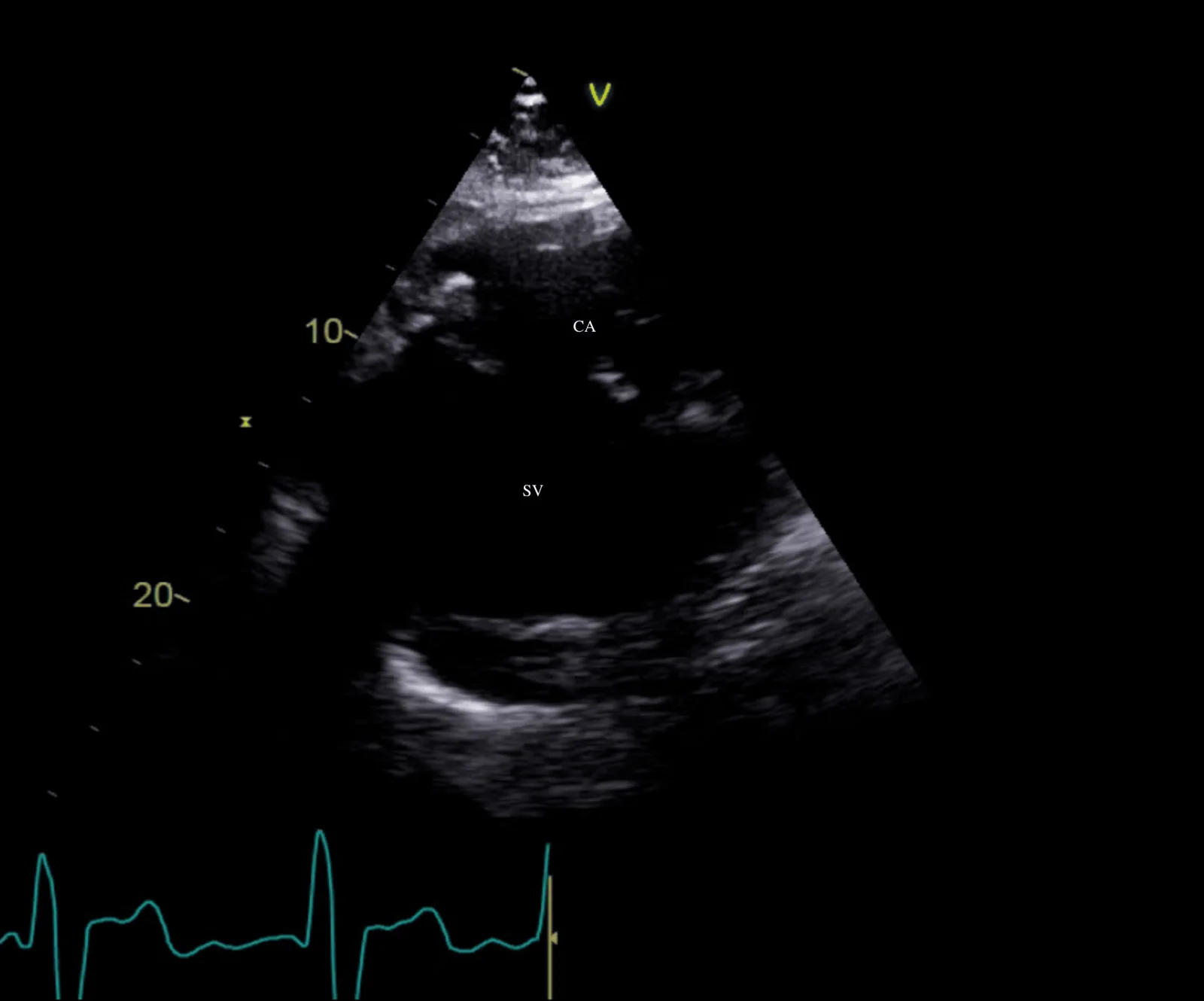
Transthoracic echocardiographic image demonstrating single-ventricle anatomy SV: single ventricular chamber, CA: common atrium

Pulmonary hypertension was clinically documented by pulmonology consultation, but formal quantification was not feasible; right ventricular systolic pressure could not be estimated due to anatomical complexity, and right heart catheterization was not performed. No pulmonary vasodilator therapy was initiated. Figure 2 shows a representative 2D grayscale echocardiographic image of the single ventricular chamber.

**Figure 3:**
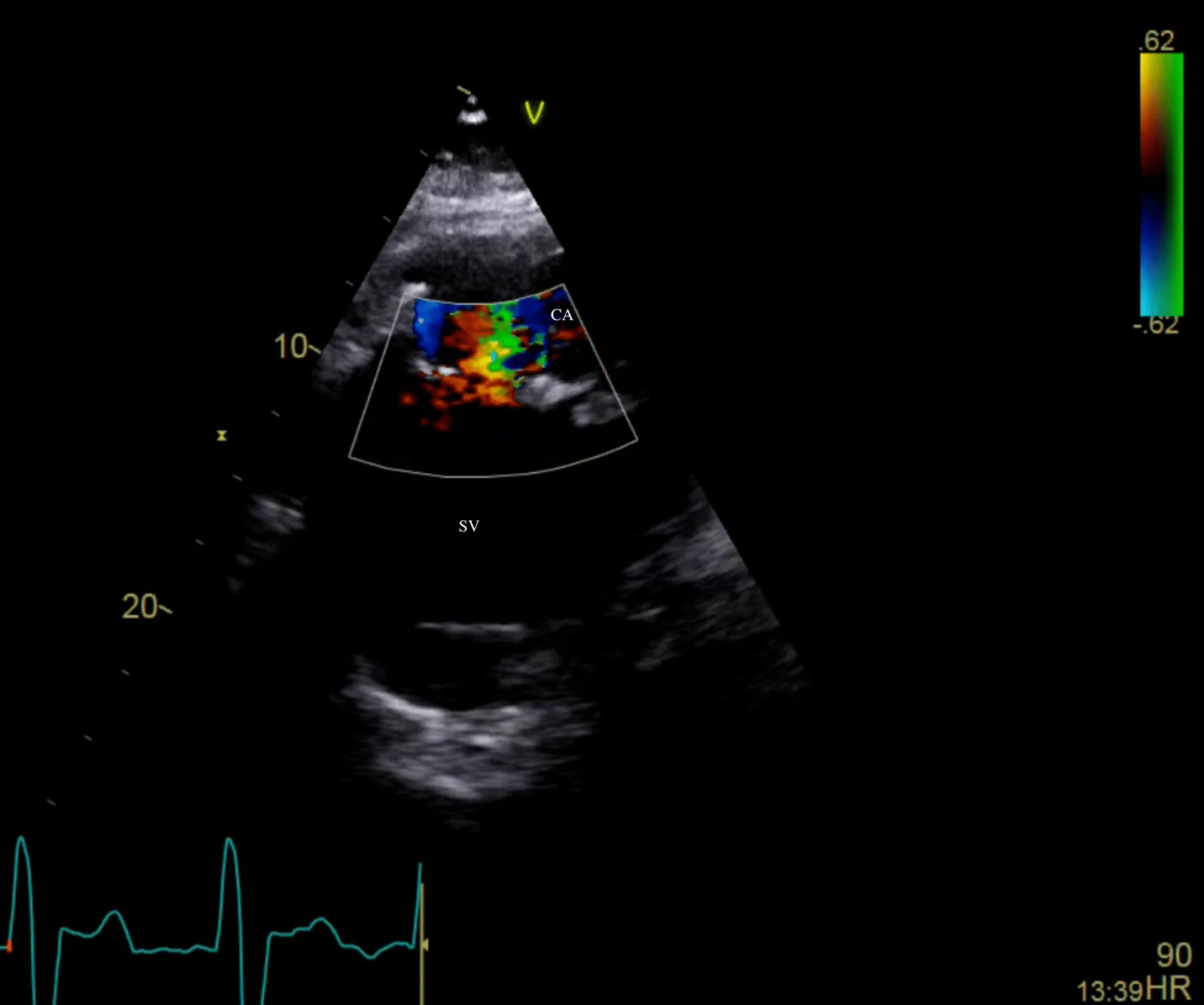
Transthoracic echocardiographic image demonstrating single-ventricle anatomy and severe common AV valve regurgitation SV: single ventricular chamber, CA: common atrium, AV: atrioventricular

Figure 3 shows color Doppler imaging demonstrating severe AV valve regurgitation.

**Figure 4:**
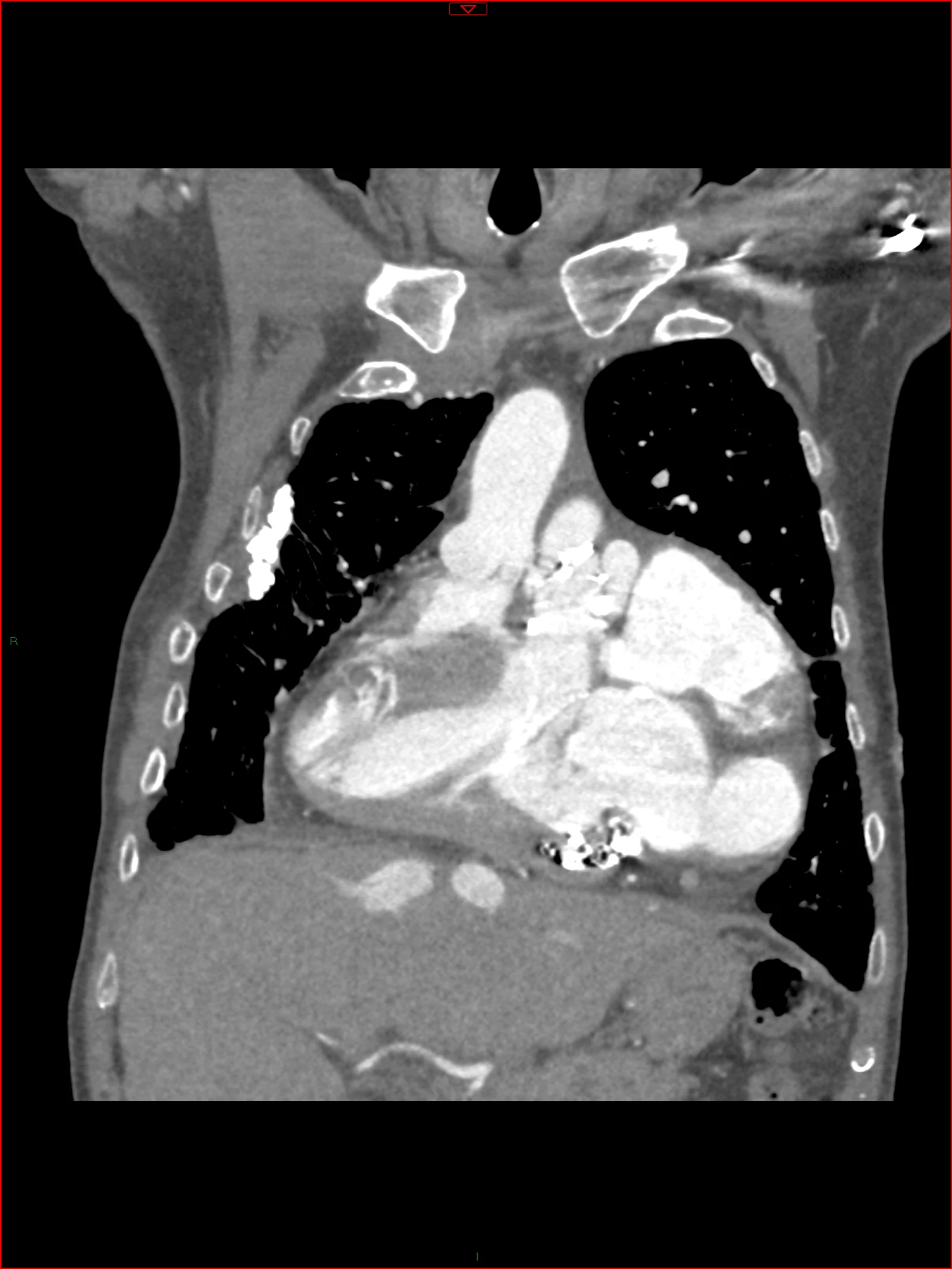
Chest CT angiography with coronal reconstruction demonstrating mesocardia and cardiomegaly CT: computed tomography

**Figure 5:**
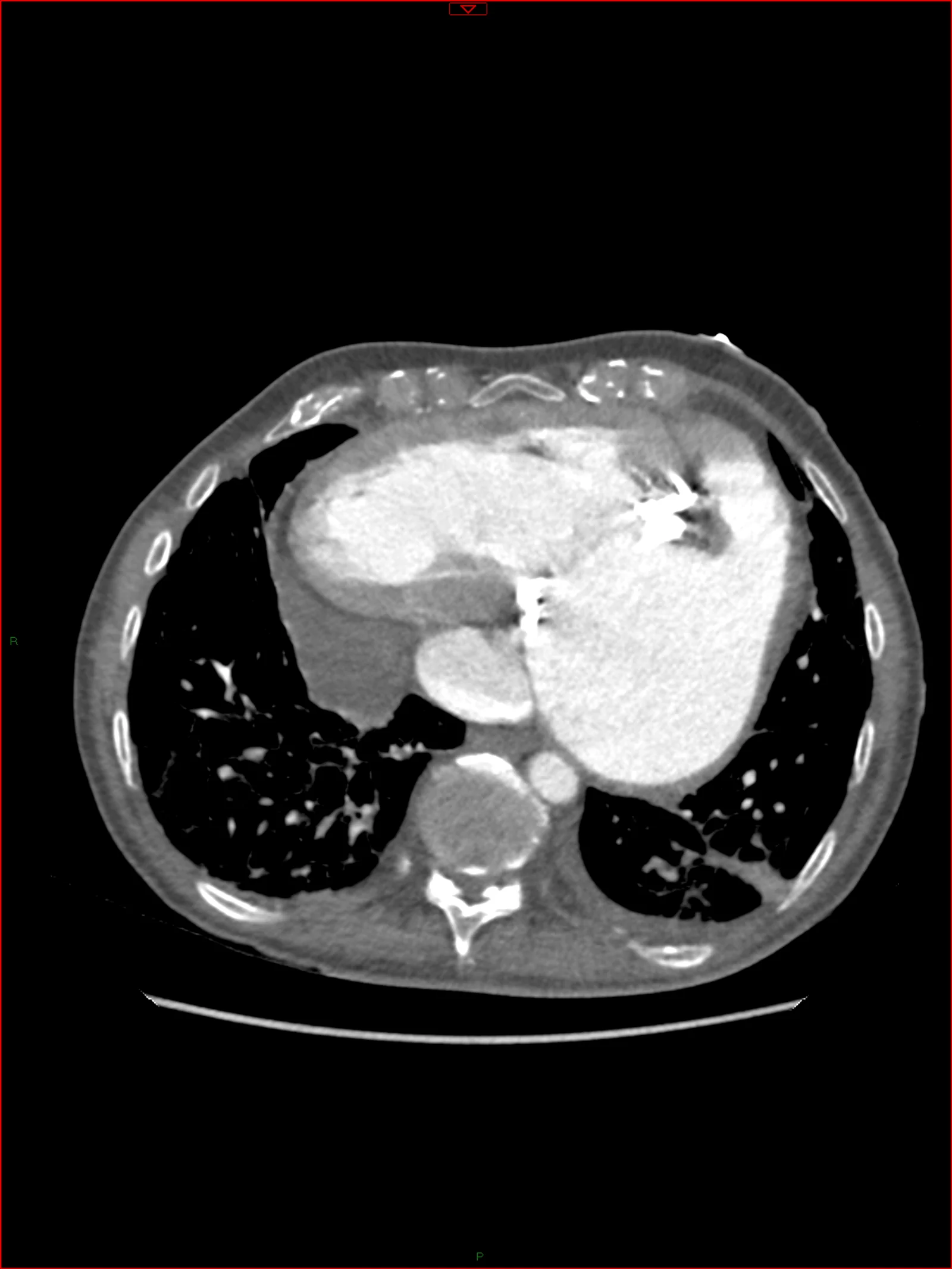
Chest CT angiography, axial image at the ventricular level demonstrating single ventricular chamber CT: computed tomography

Figures 4-5 show the coronal and axial CT images of the chest, respectively.

Video 1 shows a sequential axial CT cine loop through the thorax.

**Video 1:** Chest CT angiography showing sequential axial cine loop Sequential axial CT angiographic cine loop progressing craniocaudally. The video consists of two panels displayed side by side: the left panel shows the CT angiography window demonstrating mesocardia, a single dominant ventricular chamber without an identifiable interventricular septum, and markedly abnormal cardiac morphology consistent with single-ventricle CHD; the right panel shows the lung window demonstrating right-sided pleural thickening and volume loss from the prior right lower lobe lobectomy, as well as bilateral pleural effusions and subsegmental atelectasis consistent with the infectious process. CT: computed tomography, CHD: congenital heart disease

Management

Management targeted three concurrent problems: rate control, infection, and volume status. Bisoprolol 2.5 mg twice daily and digoxin 0.25 mg once daily were reinstated to control ventricular rate in AF [4]. Intravenous ceftriaxone 2 g daily and azithromycin 500 mg daily were initiated for community-acquired pneumonia, consistent with Infectious Diseases Society of America/American Thoracic Society (IDSA/ATS) guidelines [5]. Furosemide 40 mg intravenously once daily was administered for volume management; acenocoumarol was continued with daily INR monitoring.

Oxygen was delivered via HFNC, targeting SpO₂ 75-80%, an individualized goal reflecting chronic physiological adaptation, per 2025 ACHD guidelines [6]. Nebulized bronchodilators and inhaled budesonide were added. Ventricular rate was reduced to the 70s within 24 hours of therapy reinstatement.

Outcome and follow-up

Initial improvement was observed by hospital day 3: SpO₂ recovered to 80-84% on HFNC, and dyspnea was reduced. However, repeated HFNC weaning attempts failed across subsequent days, with SpO₂ dropping to 70% or below on any reduction in support, indicating progressive refractory hypoxemic respiratory failure.

By hospital day 9, refractory hypoxemic respiratory failure was evident. Following a goals-of-care discussion, comfort-focused care was agreed upon. He died of refractory hypoxemic respiratory failure secondary to presumed bacterial pneumonia. He was 62 years old.

## Discussion

Extreme survival in single-ventricle physiology without documented successful surgical palliation

Survival without Fontan palliation into the seventh decade is rare, especially with systemic right ventricular morphology, which is associated with worse outcomes than left ventricular morphology [7]. Most unpalliated adults with a systemic right ventricle do not survive beyond the fifth decade [1-3,7]; this patient represents an extreme outlier sustained by an inferred near-balanced pulmonary-to-systemic blood flow (estimated clinically from decades of survival without catastrophic cyanotic decompensation rather than by direct measurement), likely maintained by naturally restricted pulmonary outflow, yet demonstrating all major adverse prognostic factors: severe AV valve regurgitation, renal impairment, and probable conduction abnormality (left bundle branch block pattern on ECG) [1,2].

**Figure 6:**
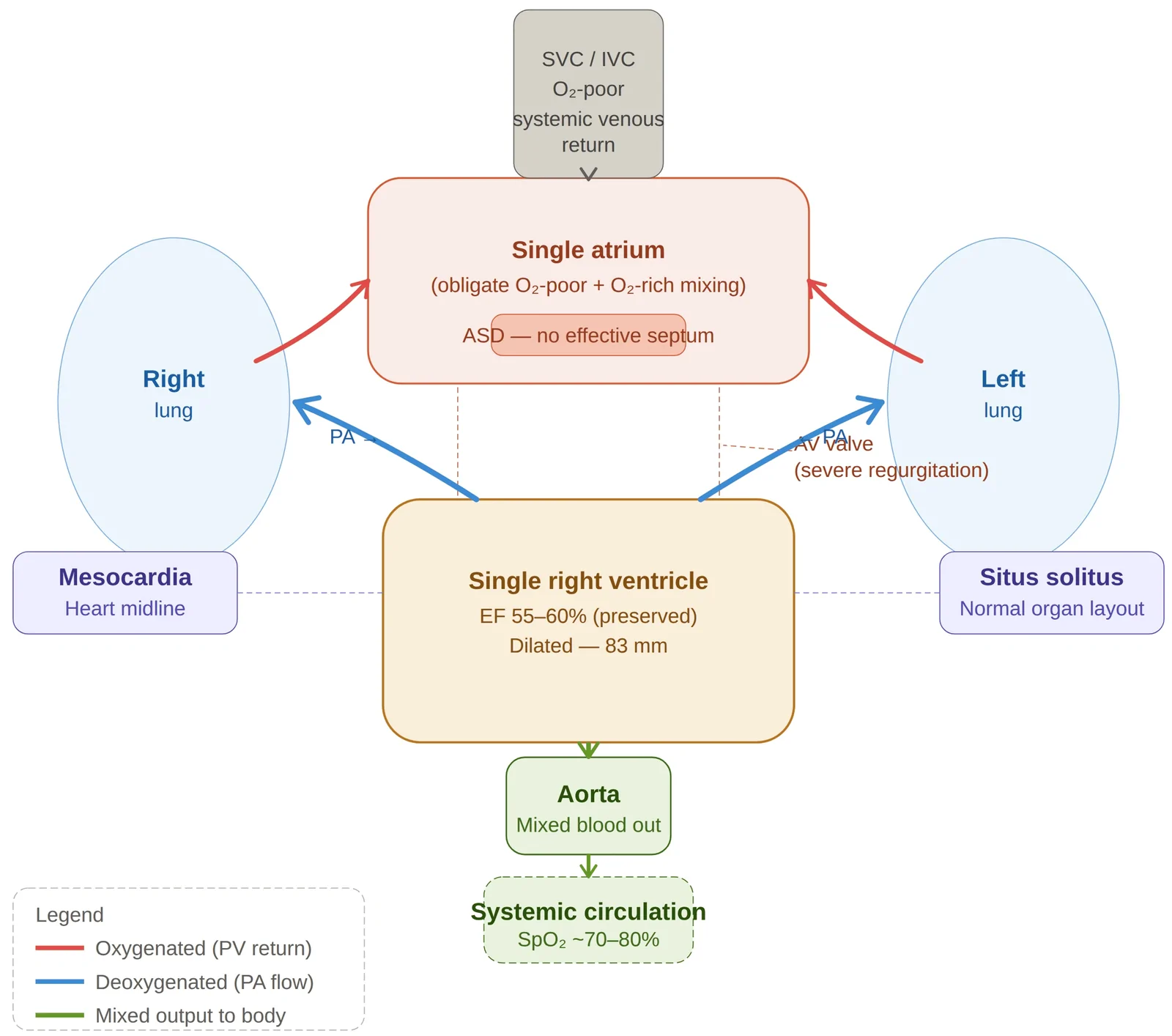
Schematic representation of the cardiac anatomy of the reported patient based on echocardiographic documentation ASD: atrial septal defect, AV: atrioventricular valve, EF: ejection fraction, IVC: inferior vena cava, PA: pulmonary artery, SpO₂: peripheral oxygen saturation, SVC: superior vena cava Image created by the authors as a schematic diagram. SVG source code was developed with AI-assisted programming (Claude, Anthropic, San Francisco, CA, USA) and rendered using a headless Chromium browser (Playwright, Microsoft Corp., Redmond, WA, USA). The figure represents original author-created content based on the echocardiographic findings of the reported patient.

Figure 6 illustrates the cardiac anatomy of this patient based on echocardiographic documentation, highlighting the key structural features: a common atrium with obligate mixing of systemic and pulmonary venous return, a single systemic right ventricle with preserved EF, and severe common AV valve regurgitation. Outflow tract anatomy, great artery relationships, and coronary anatomy could not be characterized on available imaging due to technical limitations inherent to this complex anatomy.

Preserved EF as an inadequate prognostic surrogate

This case illustrates that preserved EF may not reflect functional reserve in single-ventricle physiology, a finding not explicitly addressed in current guidelines. On the 2024 echocardiogram, EF was formally documented at 55-60%; the 2010 and 2011 studies described preserved systolic function without reporting a specific numerical EF value. The largest ventricular chamber diameter was 83 mm by 2D echocardiography (2010). The patient was NYHA Class IV, oxygen-dependent at rest, unable to ambulate without desaturation, and unable to lie flat.

In this patient, the dissociation likely resulted from severe AV valve regurgitation and loss of atrial-dependent filling, which reduced effective forward output. Furthermore, obligate right-to-left shunting may render oxygen delivery a complex function of hemoglobin, saturation, and cardiac output, rather than EF alone.

The 2025 American College of Cardiology/American Heart Association ACHD guideline [6] and the AHA noncardiac complications statement [8] acknowledge the absence of validated prognostic surrogates in adults with single ventricles; this case illustrates this clinical gap. Staging in single-ventricle physiology may benefit from integrating AV valve competence, filling pressures, and arrhythmia burden alongside EF [6,8,9]. If pulmonary hypertension was present, it may have further contributed to the hemodynamic burden; however, pulmonary pressures could not be quantified without right heart catheterization, and no vasodilator therapy could be initiated without formal pressure measurement. Hypoxemia was managed with individualized SpO₂ targets; polycythemia was managed conservatively, as compensatory erythrocytosis maintained oxygen delivery.

Arrhythmia and infection: modifiable tipping points

AF with RVR was the primary decompensating event in three of four admissions. Without effective atrial contribution to ventricular filling, rapid ventricular rates catastrophically reduce preload and effective cardiac output [10]. Rate control was pharmacologically achievable on each occasion; HR reduced from 160-180 bpm to the 70s with intravenous digoxin and bisoprolol [4], but medication nonadherence was a documented precipitant in at least two admissions. The challenge was adherence, not pharmacological response.

Clinically suspected community-acquired pneumonia was the primary diagnosis in admissions 1 and 4. Admissions 2 and 3 were primarily driven by AF with RVR and hemodynamic decompensation; an infectious etiology was considered in Admission 3, but antibiotics were deferred given the absence of radiological consolidation. Structural lung disease from the prior lobectomy, chronic pulmonary congestion, and hypoxia-mediated immune dysregulation may have collectively impaired pulmonary host defense [5]. Despite guideline-concordant antibiotic therapy, the final admission demonstrated that pneumonia superimposed on limited physiological reserve was fatal. Pneumococcal and influenza vaccination, along with early empiric treatment, are the highest-yield preventive strategies.

## Conclusions

This case illustrates that EF may be an insufficient prognostic surrogate in the context of single-ventricle physiology without documented successful surgical palliation. In this patient, AV valve regurgitation, impaired diastolic filling, and preload dependence may have collectively decoupled systolic function from effective cardiac output and functional status. In this population, prognosis may be better assessed by forward flow, preload stability, and arrhythmia burden alongside EF.
